# Emerging Biomarkers in Metabolic Dysfunction-Associated Steatotic Liver Disease (MASLD): Current Evidence, Clinical Utility, and Future Perspectives

**DOI:** 10.7759/cureus.117033

**Published:** 2026-09-27

**Authors:** Sushant S Dhanavade, Mandakini S Kshirsagar, Axita Vani, Altaf K Mujawar, Pankaj V Bansode

**Affiliations:** 1 Biochemistry, Krishna Institute of Medical Sciences, Krishna Vishwa Vidyapeeth (Deemed to Be University), Karad, IND

**Keywords:** adipokines, biomarkers, cytokeratin-18, fgf-21, liver fibrosis, masld, metabolomics, nafld, steatohepatitis

## Abstract

Metabolic dysfunction-associated steatotic liver disease (MASLD) is the most prevalent chronic liver disease worldwide and represents the hepatic manifestation of metabolic syndrome. The disease spectrum ranges from simple steatosis to steatohepatitis, fibrosis, cirrhosis, and hepatocellular carcinoma. Liver biopsy remains the histological reference standard for assessing steatohepatitis and microscopic disease activity and is valuable in selected cases; however, contemporary MASLD management increasingly relies on validated non-invasive tests for fibrosis risk stratification, with biopsy generally reserved for diagnostic uncertainty, discordant non-invasive findings, or situations in which histological information would alter management. This review aims to summarize the biochemical basis, pathophysiological mechanisms, current evidence, clinical utility, and limitations of emerging non-invasive biomarkers for the diagnosis, staging, and longitudinal monitoring of MASLD. A comprehensive narrative review of recent advances in molecular biology, metabolomics, proteomics, and inflammatory pathway research was conducted, synthesizing evidence on promising circulating biomarkers and their role in reflecting hepatic steatosis, lipotoxicity, apoptosis, inflammation, and fibrogenesis. This narrative review was conducted through a structured literature search of PubMed/MEDLINE, Scopus, and Google Scholar. Search terms included combinations of ‘metabolic dysfunction-associated steatotic liver disease,’ ‘MASLD,’ ‘MASH,’ ‘non-invasive biomarkers,’ ‘cytokeratin-18,’ ‘FGF-21,’ ‘adipokines,’ ‘cytokines,’ ‘extracellular vesicles,’ ‘microRNAs,’ ‘metabolomics,’ ‘gut microbiota,’ ‘fibrosis biomarkers,’ and ‘non-invasive liver tests.’ Priority was given to human studies, systematic reviews, meta-analyses, multicenter validation studies, and major professional-society guidelines relevant to the diagnosis, fibrosis risk stratification, prognosis, and treatment-response monitoring of MASLD. Reference lists of relevant articles were additionally reviewed to identify pertinent studies. As this was a narrative rather than a systematic review, no formal meta-analysis or standardized risk-of-bias assessment was performed. Recent research has identified several promising biomarkers, including cytokeratin-18 fragments, fibroblast growth factor-21 (FGF-21), adipokines, inflammatory cytokines, extracellular vesicles, circulating microRNAs, metabolomic signatures, and gut microbiota-derived metabolites. These biomarkers have demonstrated potential not only for diagnosing MASLD and distinguishing steatohepatitis from simple steatosis but also for predicting disease progression and monitoring therapeutic responses. However, no single biomarker currently meets all criteria to universally replace liver biopsy. Integrated multi-marker panels, enhanced by genetic data and artificial intelligence-driven algorithms, represent the most pragmatic strategy moving forward, with the aspirational goal of achieving a non-invasive, blood-based "virtual liver biopsy" in routine clinical practice.

## Introduction and background

Metabolic dysfunction-associated steatotic liver disease (MASLD), formerly known as non-alcoholic fatty liver disease (NAFLD), has emerged as a global public health challenge, affecting approximately 25-30% of the adult population worldwide [1]. The renaming to MASLD reflects a deeper understanding of its pathogenesis, emphasizing the central role of cardiometabolic risk factors and moving away from a diagnosis of exclusion [2]. MASLD encompasses a histological spectrum ranging from isolated hepatic steatosis (steatotic liver disease) to metabolic dysfunction-associated steatohepatitis (MASH), which is characterized by hepatocyte injury, lobular inflammation, and ballooning. MASH can progress to advanced fibrosis, cirrhosis, and ultimately hepatocellular carcinoma (HCC), now representing one of the fastest-growing indications for liver transplantation [3].

Despite its immense burden, the diagnosis and longitudinal monitoring of MASLD remain suboptimal. This narrative review critically evaluates emerging circulating biomarkers across four major clinical domains: identification of NASH, assessment and risk stratification of fibrosis, monitoring of therapeutic response, and prediction of disease progression and liver-related outcomes, with particular emphasis on their current level of clinical validation. Liver biopsy, while capable of distinguishing simple steatosis from NASH and quantifying fibrosis, is invasive, costly, subject to sampling error and inter-observer variability, and carries a small but non-negligible risk of complications [4]. Non-invasive imaging techniques such as ultrasound-based elastography and magnetic resonance imaging-proton density fat fraction (MRI-PDFF) accurately quantify steatosis and fibrosis but are less reliable for diagnosing MASH and are not universally accessible. MRI-PDFF provides accurate quantitative assessment of hepatic steatosis, whereas fibrosis is more appropriately assessed using liver stiffness-based techniques such as vibration-controlled transient elastography or magnetic resonance elastography [5]. Serum biomarkers offer an attractive, scalable, and repeatable alternative. Traditional liver enzymes (alanine aminotransferase, aspartate aminotransferase) lack sensitivity and specificity, as many patients with histologically advanced disease have normal transaminase levels [6]. Composite scores such as the Fibrosis-4 (FIB-4) index and the NAFLD Fibrosis Score (NFS) are validated for excluding advanced fibrosis but have a high indeterminate zone and limited utility in tracking disease dynamics [7].

The molecular era has unveiled a host of circulating molecules that reflect the complex interplay of hepatic steatosis, lipotoxicity, oxidative stress, hepatocyte apoptosis, inflammation, and fibrogenesis underlying MASLD. These emerging biomarkers, spanning cytokeratin-18 fragments, hepatokines, adipokines, inflammatory cytokines, extracellular vesicles, microRNAs, metabolomic signatures, and gut-derived metabolites, hold promise for improving diagnostic accuracy, predicting prognosis, and monitoring therapeutic responses. This review critically appraises the biochemical foundations, current evidence, clinical applicability, and future directions of these novel biomarkers in MASLD.

## Review

Pathophysiology of MASLD: a framework for biomarker development

MASLD develops from an imbalance between hepatic lipid acquisition (increased de novo lipogenesis and adipose tissue lipolysis) and disposal (fatty acid oxidation and triglyceride export) in the context of insulin resistance and adipose tissue dysfunction [8]. The ‘multiple parallel hits’ hypothesis provides a useful conceptual framework rather than a single established causal pathway. Contemporary models of MASLD pathogenesis emphasize dynamic interactions among insulin resistance, lipid dysregulation and lipotoxicity, mitochondrial and endoplasmic reticulum stress, inflammatory signaling, genetic susceptibility, gut-liver interactions, and crosstalk among hepatocytes, adipose tissue, immune cells, and hepatic stellate cells [9]. The resultant sterile inflammation, orchestrated by the nucleotide-binding oligomerization domain-like receptor protein 3 (NLRP3) inflammasome, toll-like receptors, and chemokine networks, drives the transition from steatosis to steatohepatitis. Perpetuation of injury activates hepatic stellate cells (HSCs), leading to extracellular matrix deposition and progressive fibrosis [10]. Concurrently, alterations in gut permeability, microbiota composition, and bile acid metabolism contribute to hepatic inflammation via the gut-liver axis [11]. Each of these pathogenic steps releases or generates measurable molecules into the circulation, forming the rationale for biomarker discovery (Figure 1).

**Figure 1:**
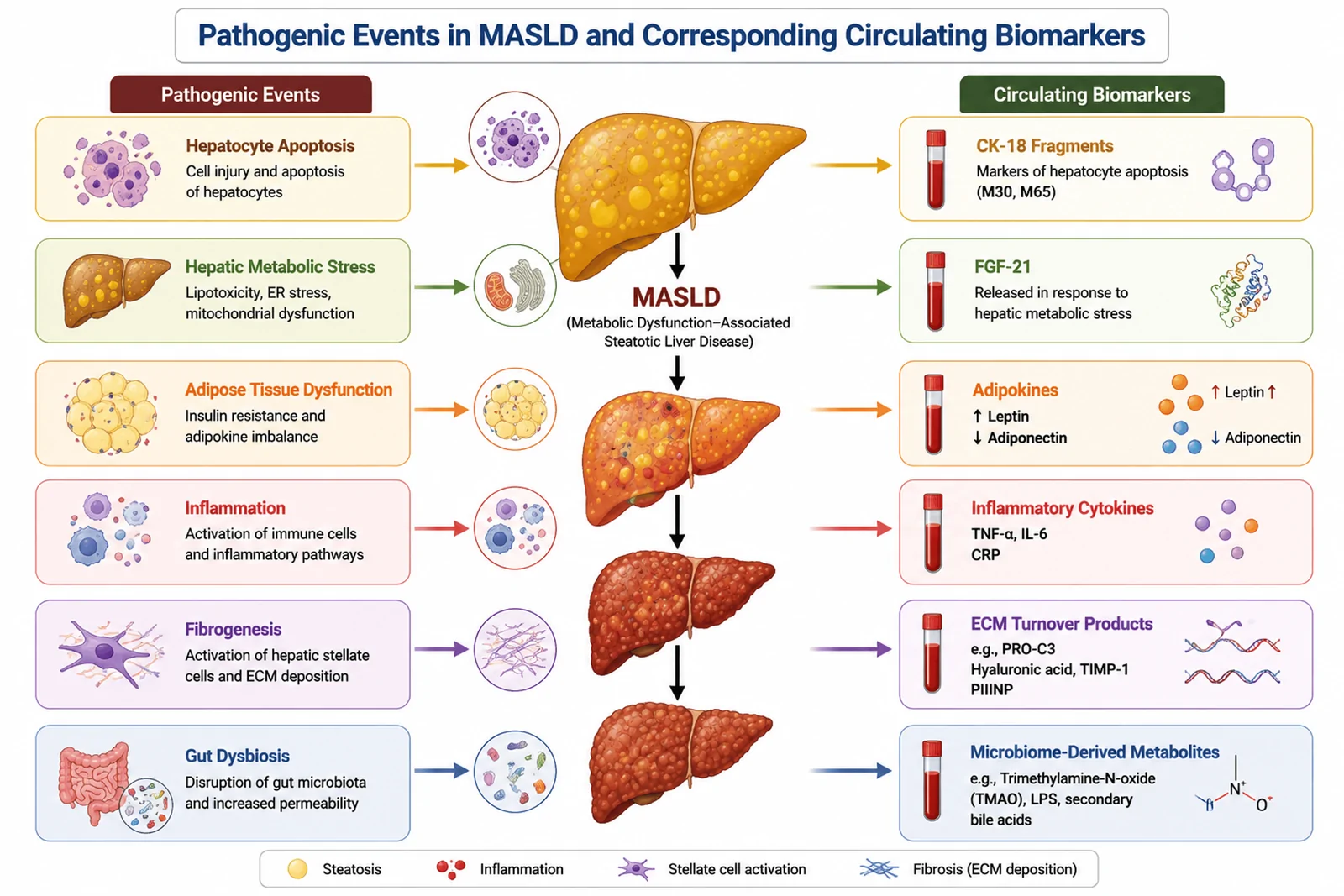
Conceptual illustration of biomarker origins in MASLD. This schematic depicts the key pathogenic events in metabolic dysfunction‑associated steatotic liver disease (MASLD) and their corresponding circulating biomarkers. Hepatocyte apoptosis releases cytokeratin‑18 (CK‑18) fragments; hepatic metabolic stress upregulates fibroblast growth factor‑21 (FGF‑21); adipose tissue dysfunction alters adipokine secretion (leptin, adiponectin); inflammation generates tumour necrosis factor‑α (TNF‑α), interleukin‑6 (IL‑6), and C‑reactive protein (CRP); fibrogenesis produces extracellular matrix turnover products (e.g., PRO‑C3); and gut dysbiosis generates microbiome‑derived metabolites such as trimethylamine‑N‑oxide (TMAO). The figure is original and created by the authors using Adobe Photoshop.

Emerging biomarkers

Cytokeratin-18 (CK-18)

CK-18 is the major intermediate filament protein of hepatocytes. During apoptosis, caspases cleave CK-18, releasing caspase-cleaved fragments (CK-18 M30), whereas during necrosis, full-length CK-18 (M65) is released. Thus, the M30:M65 ratio may distinguish apoptotic from necrotic cell death [12]. In MASH, ballooned hepatocytes and excessive apoptosis generate elevated circulating M30 levels. Numerous studies have demonstrated that CK-18 M30 can differentiate MASH from simple steatosis with an area under the receiver operating characteristic curve (AUROC) of 0.75-0.82, and levels correlate with histological activity scores [13,14]. A meta-analysis of 11 studies reported a pooled sensitivity of 75% and specificity of 70% for MASH diagnosis at a threshold of 200-250 U/L [15]. CK-18 fragments also decline with effective therapeutic interventions, including lifestyle modification, pioglitazone, and bariatric surgery [16]. However, CK-18 levels are influenced by liver diseases of other etiologies, as well as by acute alcohol intake, drug-induced liver injury, and systemic inflammatory conditions, limiting specificity. The lack of international assay standardization and variable cut-offs across populations further impede routine clinical deployment [17]. Combining CK-18 with other markers, such as FGF-21 or clinical parameters, may enhance diagnostic performance. Overall, CK-18 should be regarded as a moderately accurate adjunctive biomarker rather than a standalone diagnostic test. Reported performance varies substantially according to the CK-18 assay used (M30 or M65), analytical platform, diagnostic threshold, study population, disease prevalence, and histological reference criteria, limiting the generalizability of a single universal cut-off [18].

Fibroblast Growth Factor-21 (FGF-21)

FGF-21 is a hepatokine induced by peroxisome proliferator-activated receptor-α (PPARα) and the unfolded protein response during metabolic stress. It acts on adipose tissue, pancreas, and the central nervous system to regulate glucose and lipid metabolism, energy expenditure, and insulin sensitivity [19]. In the setting of hepatic steatosis and endoplasmic reticulum stress, FGF-21 expression is markedly upregulated, leading to elevated serum levels that correlate with intrahepatic triglyceride content. In patients with MASLD, FGF-21 concentrations increase in a stepwise manner from controls to steatosis and further to MASH [20]. A large cross-sectional study demonstrated that serum FGF-21 identified MASH with an AUROC of 0.84, outperforming CK-18 [21]. Furthermore, FGF-21 levels correlate with histological fibrosis stage and are predictive of long-term liver-related outcomes [22]. Importantly, FGF-21 analogues (e.g., efruxifermin, pegozafermin) are being developed as therapeutic agents for MASH, and circulating FGF-21 could serve as a pharmacodynamic biomarker of target engagement [23]. Despite its promise, FGF-21 is not entirely liver-specific; elevated levels are observed in obesity, type 2 diabetes, chronic kidney disease, and heart failure. Consequently, its diagnostic accuracy is attenuated in populations with a high burden of metabolic comorbidities, necessitating the use of adjusted reference ranges.

Adipokines

Adipokines, secreted predominantly by white adipose tissue, are key mediators of the crosstalk between adipose tissue and the liver. In the obese, insulin-resistant state, adipocyte hypertrophy and hypoxia lead to a pro-inflammatory shift in the adipokine profile [24].

Adiponectin

This insulin-sensitizing, anti-inflammatory, and anti-fibrotic adipokine exists as high-molecular-weight (HMW) and low-molecular-weight isoforms. HMW adiponectin is considered the most biologically active form. Hypoadiponectinemia is a consistent finding in MASLD and is independently associated with the histological severity of steatosis, inflammation, and fibrosis [25]. A prospective study showed that low total and HMW adiponectin levels predicted the development of MASLD and progression to advanced fibrosis over five years [26]. Adiponectin rises following weight loss, thiazolidinedione therapy, and bariatric surgery, paralleling histological improvement [27]. However, the modest absolute changes, sex differences, and wide inter-individual variability limit its standalone diagnostic value; it is best utilized in composite models.

Leptin

Leptin is a pro-inflammatory, profibrogenic adipokine that promotes HSC activation and hepatic fibrogenesis. Hyperleptinemia, characteristic of leptin resistance in obesity, correlates positively with hepatic steatosis and is elevated in MASH compared to simple steatosis [28]. The leptin-to-adiponectin ratio (L/A ratio) may better reflect adipose tissue dysfunction and has shown AUROCs of 0.80-0.85 for identifying NASH and significant fibrosis [29].

Other Adipokines

Resistin, visfatin, and retinol-binding protein-4 have been investigated with conflicting results and limited incremental value over established adipokines. Chemerin and omentin-1 are emerging as potential markers, but their role requires further validation [30].

Inflammatory Cytokines

The chronic low-grade inflammation characteristic of MASLD results in elevated circulating levels of pro-inflammatory cytokines. Tumor necrosis factor-alpha (TNF-α) and interleukin-6 (IL-6) are the most extensively studied. Serum TNF-α levels are higher in patients with MASH versus simple steatosis and correlate with necroinflammatory grade and fibrosis stage [31]. However, their short half-life, pulsatile secretion, and ubiquitous expression in systemic inflammatory states render them non-specific. Interleukin-1 receptor antagonist and IL-18, linked to inflammasome activation, show better discriminatory capacity for MASH in some cohorts [32]. High-sensitivity C-reactive protein, a downstream marker of IL-6-mediated hepatic acute-phase response, is consistently associated with histological severity and incident MASLD. While unlikely to replace liver-specific markers, cytokines may complement other biomarkers in multimarker panels to gauge inflammatory activity [33].

Growth differentiation factor-15 (GDF-15), a stress-responsive cytokine of the transforming growth factor-β superfamily, has recently drawn attention. Serum GDF-15 is markedly elevated in MASH and advanced fibrosis, and its levels decline with diet-induced weight loss and pharmacotherapy [34]. GDF-15 agonists (e.g., survodutide) are under clinical investigation, and GDF-15 holds dual potential as a biomarker and therapeutic target.

Extracellular Vesicles (EVs)

EVs are membrane-bound nanoparticles released by virtually all cell types, carrying proteins, lipids, mRNAs, and non-coding RNAs that reflect the state of the parent cell. Hepatocytes, Kupffer cells, and HSCs release EVs into the circulation in response to lipotoxic stress and injury [35]. The concentration, size distribution, and molecular cargo of circulating EVs are altered in MASLD. Platelet- and hepatocyte-derived EVs are increased in MASH and correlate with histological severity [36]. Proteomic analysis of EVs has identified signatures enriched in inflammatory and fibrogenic pathways that distinguish MASH from simple steatosis with high accuracy (AUROC >0.90 in pilot studies) [37]. Moreover, EV-associated microRNAs (e.g., miR-122, miR-192) show diagnostic promise [38]. Standardization of EV isolation (ultracentrifugation, size-exclusion chromatography, microfluidics) and characterization remains a major hurdle, and large-scale clinical validation is lacking. Nonetheless, EVs represent a liquid biopsy that may capture the multicellular hepatic pathology in MASLD.

Circulating MicroRNAs (miRNAs)

miRNAs are small non-coding RNAs that post-transcriptionally regulate gene expression. miR-122 is the most abundant miRNA in hepatocytes, constituting approximately 70% of the total hepatic miRNA pool, and plays a critical role in lipid metabolism, hepatic differentiation, and hepatocarcinogenesis [39]. Circulating miR-122 levels are significantly elevated in MASLD and correlate with steatosis grade and histologic activity, declining in response to weight loss and ursodeoxycholic acid treatment [40]. Other liver-enriched miRNAs, including miR-192, miR-34a, and miR-375, are dysregulated in MASH and fibrosis. A panel comprising miR-122, miR-192, miR-21, and miR-34a yielded an AUROC of 0.85 for differentiating MASH from steatosis in a multicenter study [41].

Beyond diagnosis, circulating miRNAs may predict disease progression. miR-122 and miR-21 are pro-fibrotic, while the miR-29 family acts as anti-fibrotic regulators; their ratios correlate with collagen deposition [42]. Challenges include hemolysis-induced variability, poor inter-platform reproducibility, and the lack of standardized normalization methods. Nonetheless, ongoing efforts in pre-analytical optimization and cross-platform calibration may soon enable robust miRNA-based tests.

Metabolomic Signatures

Metabolomics enables comprehensive profiling of small-molecule metabolites (<1.5 kDa) in biofluids, reflecting integrated genetic, environmental, and microbial influences on hepatic metabolism. Mass spectrometry and nuclear magnetic resonance spectroscopy-based studies have identified reproducible metabolic perturbations in MASLD: increased branched-chain amino acids (BCAAs: valine, leucine, isoleucine), aromatic amino acids (phenylalanine, tyrosine), glutamate, and lactate; decreased glycine, serine, and glutamine; and alterations in bile acids, phospholipids, and acylcarnitines [43,44]. Glutamate, isoleucine, and specific triacylglycerol species have been correlated with liver fat content and MASH on biopsy [45].

A lipidomics-based model (OWLiver) that incorporates individual triglyceride species demonstrated an AUROC of 0.95 for MASH diagnosis in a discovery cohort, though validation is ongoing [46]. Bile acid profiling has revealed that an elevated ratio of conjugated primary to secondary bile acids, reflecting gut dysbiosis and altered farnesoid X receptor (FXR) signaling, is associated with MASH and advanced fibrosis [47]. Serum metabolites also track therapeutic response; for instance, a decrease in BCAA levels accompanies histological improvement after bariatric surgery [48]. The main limitations are high analytical costs, need for specialized equipment, batch effects, and the challenge of translating complex multivariate patterns into clinically actionable single-score tests. Ongoing initiatives such as the LITMUS (Liver Investigation: Testing Marker Utility in Steatohepatitis) consortium have undertaken large-scale validation of non-invasive biomarkers for steatohepatitis and fibrosis across multicenter cohorts [49].

Gut Microbiota-Derived Metabolites

The gut microbiota modulates host metabolism through production of short-chain fatty acids (SCFAs), bile acid metabolism, choline conversion, and ethanol generation. Dysbiosis in MASLD is characterized by increased Bacteroidetes/Firmicutes ratio, small intestinal bacterial overgrowth, and altered abundance of specific genera (e.g., increased Escherichia, decreased Faecalibacterium prausnitzii) [50].

Trimethylamine-N-oxide (TMAO)

Dietary phosphatidylcholine and L-carnitine are converted by gut bacteria to trimethylamine, which is oxidized in the liver to TMAO. Circulating TMAO levels are elevated in MASLD and correlate with the severity of steatosis and inflammation, and independently predict cardiovascular events in this population [51].

Endogenous Ethanol

Gut bacteria, particularly Proteobacteria and Klebsiella pneumoniae, can produce ethanol from carbohydrates. Elevated blood ethanol levels (detected by highly sensitive gas chromatography) have been reported in patients with MASH, even in the absence of alcohol consumption, reinforcing the concept of "auto-brewery syndrome" contributing to liver injury [52].

Short-Chain Fatty Acids

Acetate, propionate, and butyrate, produced by fermentation of dietary fiber, exert anti-inflammatory effects and maintain gut barrier integrity. Fecal SCFA concentrations are reduced in MASLD, and a low propionate-to-acetate ratio correlates with hepatic steatosis [53].

3-(4-Hydroxyphenyl) Lactate

3-(4-Hydroxyphenyl) lactate is a tyrosine metabolite produced by gut bacteria, this compound has been identified in a metabolome-wide association study as a strong predictor of future liver-related mortality in patients with MASH, highlighting the potential of gut-derived metabolites as prognostic biomarkers [54].

The clinical translation of microbiota-derived biomarkers faces obstacles including variability in diet, medications (especially proton pump inhibitors and antibiotics), and the complex functional redundancy within microbial communities. Multi-omics integration, combining metagenomics, metatranscriptomics, and metabolomics, may overcome some of these issues but is currently confined to research settings. A summary of the biological basis, clinical utility, and limitations of these biomarkers is presented in Table 1.

**Table 1:** Summary of principal emerging circulating biomarkers in MASLD: biological basis, clinical utility and major limitations. The table provides an overview of biomarkers discussed in the review, highlighting their pathophysiological origin, potential diagnostic or prognostic applications, and key obstacles to clinical translation. Data are synthesized from the literature and supported by meta‑analyses and large cohort studies (e.g., Musso et al. [14] and references therein). MASLD, metabolic dysfunction‑associated steatoic liver disease; MASH, metabolic dysfunction‑associated steatohepatitis; CKD, chronic kidney disease; TMAO, trimethylamine‑N‑oxide; SCFAs, short‑chain fatty acids.

Biomarker	Biological basis	Clinical utility	Major limitation
CK-18	Hepatocyte apoptosis	Differentiates MASH from simple steatosis	Limited specificity; assay variability
FGF-21	Hepatic metabolic stress	Detects MASH; predicts fibrosis	Elevated in obesity, diabetes, CKD
Adiponectin	Insulin sensitivity	Correlates with steatosis and fibrosis	High inter-individual variability
Leptin	Pro-fibrogenic adipokine	Reflects adipose dysfunction	Influenced by obesity
TNF-α/IL-6	Inflammation	Indicates inflammatory activity	Poor liver specificity
Extracellular vesicles	Cell-to-cell signaling	Potential liquid biopsy	Lack of standardized isolation
miRNAs (miR-122, miR-192)	Gene regulation	Diagnosis and disease monitoring	Standardization issues
Metabolomic signatures	Altered metabolism	High diagnostic accuracy	Expensive and technically demanding
Gut microbiota metabolites (TMAO, SCFAs)	Gut–liver axis	Disease progression and prognosis	Affected by diet and microbiome variability

Clinical utility and implementation challenges

The ultimate goal of non-invasive biomarkers is to replace or substantially reduce the need for liver biopsy in diagnosis, risk stratification, therapeutic decision-making, and monitoring treatment response. An ideal biomarker should be liver-specific, measurable by standardized high-throughput assays, biologically plausible, and validated across diverse populations with well-defined cut-offs. Presently, no single emerging biomarker meets all these criteria.

Composite models and sequential algorithms integrating clinical, genetic (e.g., PNPLA3, TM6SF2, HSD17B13 variants), and novel molecular markers hold the greatest promise. For example, the NIS4® panel (miR-34a, alpha-2 macroglobulin, YKL-40, HbA1c) accurately identifies at-risk MASH with an AUROC of 0.80 [55]. Emerging molecular biomarkers should be interpreted in the context of established non-invasive strategies. Current clinical pathways generally use FIB-4 as an accessible first-line fibrosis risk-stratification tool, followed by secondary assessment with VCTE, ELF, or other validated modalities when indicated. MRE provides highly accurate liver-stiffness assessment in selected patients, whereas MRI-PDFF quantitatively assesses hepatic fat rather than fibrosis. Compared with these established approaches, CK-18, FGF-21, circulating miRNAs, EVs, metabolomic signatures, and microbiota-derived metabolites may provide greater pathobiological specificity but currently have less standardized assays, fewer externally validated thresholds, and limited evidence of incremental benefit in routine patient management. Accordingly, their most plausible near-term role is complementary rather than substitutive. The ELF score (hyaluronic acid, TIMP-1, PIIINP) enhanced with CK-18 and clinical variables improves fibrosis detection [56]. Artificial intelligence and machine learning are being leveraged to integrate multi-dimensional data (demographics, blood tests, multi-omics, imaging radiomics) into robust diagnostic algorithms [57].

Regulatory and practical barriers must be addressed. Analytical validation of assays (precision, reproducibility, inter-laboratory concordance) and clinical validation in longitudinal, multicenter cohorts reflecting the real-world spectrum of metabolic comorbidities are essential. The dynamic range of each biomarker in response to pharmacotherapy, particularly with the imminent approval of MASH drugs (resmetirom, FGF-21 analogues, GLP-1 receptor agonists), must be established. Cost-effectiveness analyses will determine whether the added value of novel biomarkers justifies their price over existing non-proprietary indices like FIB-4. Finally, clinician education and integration into electronic health record systems are needed for successful adoption.

Future perspectives

The future of biomarker development in MASLD lies in precision medicine. Single-cell and spatial transcriptomics of liver tissue are revealing novel cell-type-specific markers that may be assayed in blood, such as shed ectodomains of HSC-specific proteins and immune cell-derived molecules [58]. Liquid biopsies encompassing circulating tumor DNA, cell-free RNA, and EV-derived analytes will allow simultaneous assessment of steatohepatitis activity, fibrosis stage, and HCC risk. The liver-adipose-gut axis will continue to be a fertile source of integrated signatures.

Large collaborative initiatives such as LITMUS and NIMBLE are evaluating and validating non-invasive biomarkers against histological reference standards and advancing selected biomarkers through regulatory qualification pathways [59,60]. The FDA-NIH BEST (Biomarkers, EndpointS, and other Tools) framework provides a pathway for biomarker qualification as drug development tools, which will accelerate their use in clinical trials and eventually clinical practice [61].

The concept of a "virtual liver biopsy," a composite blood-based score that reliably quantifies steatosis, inflammation, and fibrosis simultaneously, may soon become a reality. Such tools, complemented by point-of-care imaging and artificial intelligence interpretation, could transform MASLD management from an invasive, specialist-dependent pathway to a streamlined, primary-care-based screening and monitoring model, ultimately reducing the enormous global burden of this disease.

## Conclusions

The landscape of non-invasive biomarkers for MASLD has expanded dramatically from traditional liver enzymes and simple fibrosis scores to a sophisticated repertoire of molecules reflecting hepatic apoptosis, metabolic stress, adipose tissue dysfunction, systemic inflammation, EV signaling, epigenetic regulation, metabolic reprogramming, and gut-liver axis disruption. While CK-18, FGF-21, adipokines, and metabolomic signatures have individually demonstrated moderate-to-high accuracy for diagnosing MASH and predicting fibrosis progression, none has yet attained the performance and standardization required to replace liver biopsy universally. The integration of multiple biomarkers into multimarker panels, enhanced by genetic and clinical data, represents the most pragmatic immediate strategy. Ongoing validation efforts in large longitudinal cohorts and the emergence of pharmacodynamic biomarkers will pave the way for their implementation in routine clinical care and therapeutic trials. As precision medicine paradigms advance, a non-invasive, blood-based "virtual liver biopsy" is an aspirational yet attainable goal that promises to revolutionize MASLD diagnosis and management.

Among the emerging biomarkers reviewed, CK-18 has one of the largest clinical evidence bases but remains limited by assay and threshold heterogeneity, while FGF-21 and adipokine-based approaches require further prospective validation before routine diagnostic use. EVs, circulating miRNAs, metabolomic signatures, and gut microbiota-derived metabolites remain predominantly investigational. At present, established approaches such as FIB-4, ELF, and elastography retain stronger clinical readiness for fibrosis risk stratification. Future implementation of emerging biomarkers will require standardized assays and cut-offs, prospective multicenter external validation, demonstration of incremental value over established non-invasive tests, longitudinal association with clinically meaningful outcomes, and evidence of cost-effectiveness. Multimarker approaches are promising, but the concept of a blood-based ‘virtual liver biopsy’ should presently be regarded as an aspirational research goal rather than an established clinical tool.
